# Clostridium septicum: A usual suspect? Aortic rupture following right hemicolectomy: A case report

**DOI:** 10.1016/j.ijscr.2018.10.083

**Published:** 2018-11-22

**Authors:** Sam Cresser, Lachlan Maddock, Philip Smart

**Affiliations:** a Eastern Clinical School, Eastern Health, Melbourne Australia; b Department of Vascular Surgery, Eastern Health, Melbourne Australia; c Department of Surgery, Epworth Health, Melbourne Australia; d Gastrointestinal Clinical Institute, Epworth HealthCare, Melbourne Australia

**Keywords:** Aneurysm, Infected, Colonic neoplasm, Case report

## Abstract

**Introduction:**

There is a known association between *Clostridium Septicum*, mycotic aneurysm and colon neoplasm.

**Case presentation:**

We report the case of a 90 year old female admitted with abdominal pain to the general surgery unit. Admission CT scan demonstrated a thick walled caecum and pericaecal inflammation suspicious for a perforated carcinoma. This was subsequently confirmed at colonoscopy with biopsy demonstrating a poorly differentiated adenocarcinoma. A laparoscopic right hemicolectomy was completed the following week after perioperative workup and intravenous antibiotic therapy. On the 9th postoperative day, fever and rising inflammatory markers prompted repeat abdominal CT scan which demonstrated a mycotic aneurysm of the upper abdominal aorta. After discussion with the vascular surgery and infectious diseases team, along the patient and family, the decision was made to palliate. The patient died at home from presumed spontaneous rupture two weeks after discharge.

**Discussion:**

Mycotic aneurysm in colonic malignancy is a rare and often lethal complication. *C. Septicum* is causative in over 70% of cases with concomitant colonic malignancy.

**Conclusion:**

Mycotic aneurysm should be considered in any deteriorating patient with concomitant colonic malignancy.

## Highlights

• Mycotic aneurysm in colon cancer is a rare and lethal complication.
• *C. Septicum* is causative in over 70% of cases with associated colonic malignancy.
• Clinicians should be aware of this clinical entity and consider it in any deteriorating patient with associated colonic malignancy.
• Early antibiotics are essential however surgery remains the mainstay of treatment.

## 1 Introduction

Mycotic Aneurysm (MA) is a rare, severe variant of aortic aneurysm accounting for 0.7%–3.3% of all aortic aneurysms, and without treatment all cases are fatal [[1], [2], [3], [4]]. There is a well known association between *C. Septicum*, mycotic aneurysm and colonic malignancy. We present the case of a 90 year old woman who developed fever and general malaise post right laparoscopic hemicolectomy for caecal adenocarcinoma in a private metropolitan hospital. A mycotic abdominal aortic aneurysm was later discovered on CT scan, with the view that this infection was likely seeded from the caecal rupture. A literature review was also conducted, reviewing the association between *C. Septicum* and colonic malignancy. This case has been reported in line with the SCARE criteria [5].

## 2 Case presentation

**Fig. 1:**
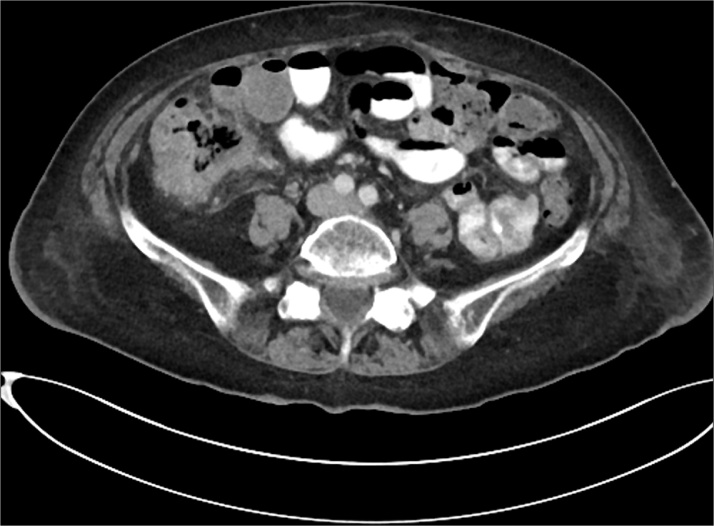
Admission CT scan demonstrating thick walled caecum with pericaecal inflammation.

A 90 year old woman was admitted to our General Surgery Unit with widespread abdominal pain after recent self discharge from another hospital with a diagnosis of caecal colitis. Past medical history was significant for normal colonoscopy two years prior to presentation, as well as appendicectomy, cholecystectomy and hysterectomy. On admission the patient underwent an abdominal/pelvic CT scan as well as basic pathology testing. Pathology results were unremarkable aside from a CRP of 65 mg/L (<5). The CT scan demonstrated a thick walled caecum and pericaecal inflammation suspicious for a perforated carcinoma (Fig. 1).

**Fig. 2:**
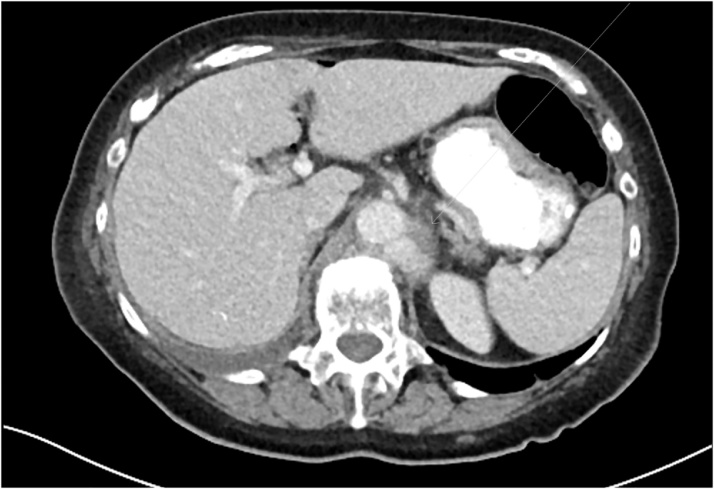
Post-operative transverse CT scan demonstrating mycotic abdominal aortic aneurysm.

**Fig. 3:**
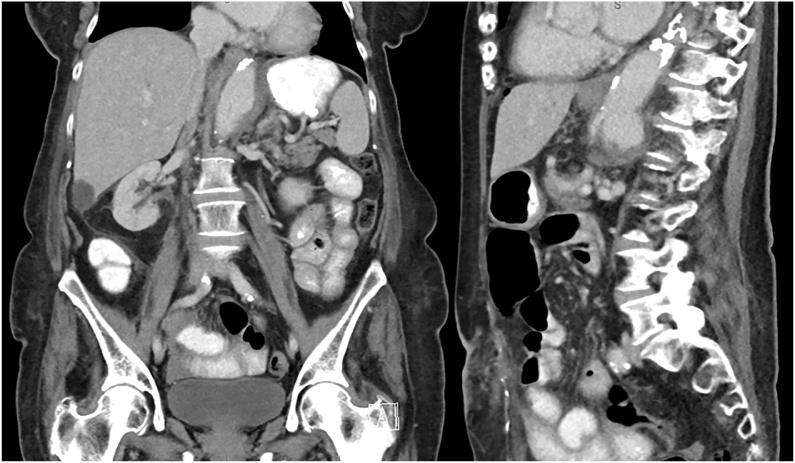
Coronal and sagittal views of the mycotic abdominal aortic aneurysm.

A colonoscopy was performed following intravenous antibiotic therapy and echocardiography. Endoscopy demonstrated an obvious neoplasm in the caecum. Histology confirmed an infiltrating poorly differentiated adenocarcinoma. Laparoscopic right hemicolectomy was performed by the consultant colo-rectal surgeon a week later. Post op recovery was uneventful. On the seventh postoperative day the patient developed low grade fever of 38 ° on the context of increasing malaise, lethargy and non-specific abdominal pain. A septic screen was performed which demonstrated a white cell count rise to 15.5 (10^9/L) and a CRP of 90 mg/L, however chest x-ray, urine culture and blood cultures all remained negative. Subsequent CT scan demonstrated a mycotic abdominal aortic aneurysm in the upper abdominal aorta involving coeliac axis and superior mesenteric artery. Tazocin was initiated and the vascular surgery team was consulted. The aneurysm was not suitable for endovascular stent due to anatomic location across major visceral arteries, and major surgery for open repair deemed inappropriate (Fig. 2, Fig. 3).

Available treatment options were discussed with the patient and family. The patient was later discharged and palliated at home, dying from presumed spontaneous aortic rupture 2 weeks later.

## 3 Discussion

William Osler first used the term mycotic aneurysm in 1885 to describe the complications of syphilitic endarteritis in a 30 year old man leading to multiple saccular aneurysms [6]. Presently, mycotic aneurysm (MA) can be as defined by (A) infectious aortitis leading to aneurysmal formation within the vessel wall or (B) infection of a pre-existing aneurysm within the vessel wall by any and all micro-organisms, the latter mechanism being the more common of the two [4]. The major aetiology of mycotic aneurysm involves bacterial seeding, either into a previous defect in the intimal wall or via septic emboli in the vasa vasorum. Contiguous spread of infection or direct inoculation via trauma such as penetrating injury have also been documented but are rare [[2], [3], [4],^7^]. Causative organisms include staphlyococcus, salmonella and some streptococcus species (A to C), with *C. Septicum* being a rare cause, responsible for only 1.3% of all clostridial infections [2,3]. Other organisms have been associated with colonic malignancy, with *Streptococcus Bovis* endocarditis being associated with concommitant colonic malignancy in 16–62% of cases, however *C. Septicum* aortitis forms a distinct clinical entity [8]. A review article by Alimi in 2017 found 51 documented cases of *C. Septicum* aortitis, with colonic malignancy or premalignant lesions identified in 71% of cases [1]. Prognosis is poor with a 57% mortality rate in *C. Septicum* vasculitis, with a 100% mortality in those who did not undergo operative treatment. In the reported case, no organism was isolated, blood cultures remained negative and an autopsy was not performed, precluding organism isolation from the tissue. Despite this, given the strong association between *C. Septicum* and mycotic aneurysm in patients with colonic malignancy, *C. Septicum* was the most probable organism.

## 4 Conclusion

Mycotic aneurysm in colonic malignancy is a rare and lethal complication with *C. Septicum* being causative in over 70% of cases with concomitant colonic malignancy. Clinicians should be aware of this clinical entity and consider it in any deteriorating patient with concomitant colonic malignancy.

## Conflict of interest

The authors have nothing to disclose.

## Funding

This study was supported by Epworth Research Institute Major Research Grant No. 11.952.000.80982. This paper is not based on a communication to a society or meeting.

## Ethical approval

This case report has been exempted from Ethics approval by Epworth Health.

## Consent

Singed consent was not able to be obtained, despite exhaustive attempts by the hospital team. The case report has been completely de-identified, so not to cause harm to the patient or their family. A signed statement to this effect provided by the Coloretal Surgeon, Department of Surgery.

## Author contribution

Sam Cresser: Data collection, writing the paper.

Lachlan Maddock: Study concept and design, writing and review of paper.

Phillip Smart: Study concept and design, writing and review paper.

## Registration of research studies

N/A.

## Guarantor

The Guarantor for this case report is Sam Cresser.

## Provenance and peer review

Not commissioned externally peer reviewed.
